# Chromosomal evolution in *Raphicerus* antelope suggests divergent X chromosomes may drive speciation through females, rather than males, contrary to Haldane's rule

**DOI:** 10.1038/s41598-021-82859-0

**Published:** 2021-02-04

**Authors:** Terence J. Robinson, Halina Cernohorska, Svatava Kubickova, Miluse Vozdova, Petra Musilova, Aurora Ruiz-Herrera

**Affiliations:** 1 Department of Botany and Zoology, Stellenbosch University, Stellenbosch, South Africa; 2 Veterinary Research Institute, Hudcova 70, Brno, Czech Republic; 3 Departament de Biologia Cel·lular, Fisiologia i Immunologia, Universitat Autònoma de Barcelona (UAB), 08193 Cerdanyola del Vallès, Spain; 4 Genome Integrity and Instability Group, Institut de Biotecnologia I Biomedicina (IBB), Universitat Autònoma de Barcelona (UAB), 08193 Cerdanyola del Vallès, Spain

**Keywords:** Evolutionary genetics, Evolutionary theory, Speciation

## Abstract

Chromosome structural change has long been considered important in the evolution of post-zygotic reproductive isolation. The premise that karyotypic variation can serve as a possible barrier to gene flow is founded on the expectation that heterozygotes for structurally distinct chromosomal forms would be partially sterile (negatively heterotic) or show reduced recombination. We report the outcome of a detailed comparative molecular cytogenetic study of three antelope species, genus *Raphicerus*, that have undergone a rapid radiation. The species are largely conserved with respect to their euchromatic regions but the X chromosomes, in marked contrast, show distinct patterns of heterochromatic amplification and localization of repeats that have occurred independently in each lineage. We argue a novel hypothesis that postulates that the expansion of heterochromatic blocks in the homogametic sex can, with certain conditions, contribute to post-zygotic isolation. i.e., female hybrid incompatibility, the converse of Haldane’s rule. This is based on the expectation that hybrids incur a selective disadvantage due to impaired meiosis resulting from the meiotic checkpoint network’s surveillance of the asymmetric expansions of heterochromatic blocks in the homogametic sex. Asynapsis of these heterochromatic regions would result in meiotic silencing of unsynapsed chromatin and, if this persists, germline apoptosis and female infertility.

## Introduction

The chromosomal speciation theory^1,2^ also referred to as the “Hybrid dysfunction model”^3^, has been one of the most intriguing questions in biology for decades. It relies on the development of chromosomal incompatibility between divergent lineages by invoking post-zygotic isolating mechanisms that lead to a point when a species eventually becomes two under a model of bifurcating evolutionary history. A common feature underlying the genetics of post-zygotic isolation is that the heterogametic sex (i.e., XY in mammals) is much more likely to be affected under Haldane's rule^4^. Proponents of this theory posit that structurally rearranged chromosomes must reduce the fitness of heterozygotes. Once fixed (i.e., homozygous) in a population (often invoking meiotic drive or drift to achieve this), these rearrangements would facilitate lineage divergence on the grounds that hybrids are expected to be at a selective disadvantage due to impaired viability or fertility^5,6^.

Although numerous studies are considered to support the hybrid disfunction model (see Brown and O’Neill^7^ for an overview of the historical context and evidence for many of the current models of chromosomal speciation), a major weakness detracts from its general acceptance. If strongly underdominant rearrangements (with potential to disrupt gene flow) can spread from an initial heterozygous state to become fixed in a population, they would intuitively not constitute strong barriers to gene flow between diverging lineages homozygous for the unaltered and altered chromosomal states. Consequently support shifted to hypotheses that do not necessarily invoke chromosomal rearrangement to reduce fitness, but serve rather to impede gene flow between populations by suppressing recombination i.e., the “Suppressed recombination models of speciation”^3,5–7^.

In other words, and in a marked departure from the hybrid dysfunction model, chromosomal rearrangements could facilitate lineage divergence in the face of continuing gene flow and that reduction of recombination between chromosomes carrying different rearrangements was the *sine qua non* for speciation^6^. This is perhaps best reasoned for instances involving inversions that permit the accumulation of incompatible alleles in regions protected from recombination, while genetic exchange in colinear segments of the rearranged chromosomes is freely permitted^8,9^. Direct and indirect evidence of suppressed recombination induced by inversions is abundant in the literature^10–14^. This contributes to a general framework for inversion-driven recombination suppression that may facilitate the accumulation of genetic incompatibilities, the so-called speciation genes^15,16^, and mutations that confer local adaptation that drive genetic divergence^17^.

A further but less explored category of chromosomal change that has gained traction as a potential cause of genomic conflict and subsequent incipient species formation entails differences in heterochromatin and the proteins involved in its epigenetic modification^7,18–21^. DNA sequences of heterochromatic regions typically display tandemly repeated DNA motifs in large arrays that rapidly diverge (due to gene conversion, replication slippage and unequal recombination^22–25^) and can accelerate karyotypic reorganization^26^.

With this as context, we explore a novel hypothesis that suggests the expansion of heterochromatic blocks in the homogametic sex may, with certain conditions (size, different sequence composition and location), contribute to post-zygotic isolation. In support of this, the molecular cytogenetic and fine-scale chromosomal relationships of three African antelope species (genus *Raphicerus*) of the Family Bovidae (antelope, cattle, sheep and goats) are described. The two most prominent aspects of bovid chromosome evolution, an emblematic mammalian group for studying the role of chromosomal rearrangements in speciation, are the high number of autosomal Robertsonian (Rb) fusions that reflect in species’ diploid numbers (from 2n = 30 to 2n = 60) and pronounced X chromosome variation^27–29^. The latter includes the disruption of highly conserved euchromatic regions by centromere repositioning, autosomal translocation and, the focus of this paper, heterochromatic variation observed in defined regions of these chromosomes. We evaluate whether differences in the amount and distribution of non-centromeric heterochromatin on the *Raphicerus* X chromosomes permit insights into possible lineage divergence—one mediated not by the heterogametic sex (the conventional expectation) but by female meiosis and thus the converse of Haldane’s rule. We hypothesise that failure to establish reasonably persistent synapsis of the X chromosomes during first meiotic division of heteromorphic hybrids would result in a selective disadvantage that facilitates divergence among lineages.

## Results and discussion

**Figure 1:**
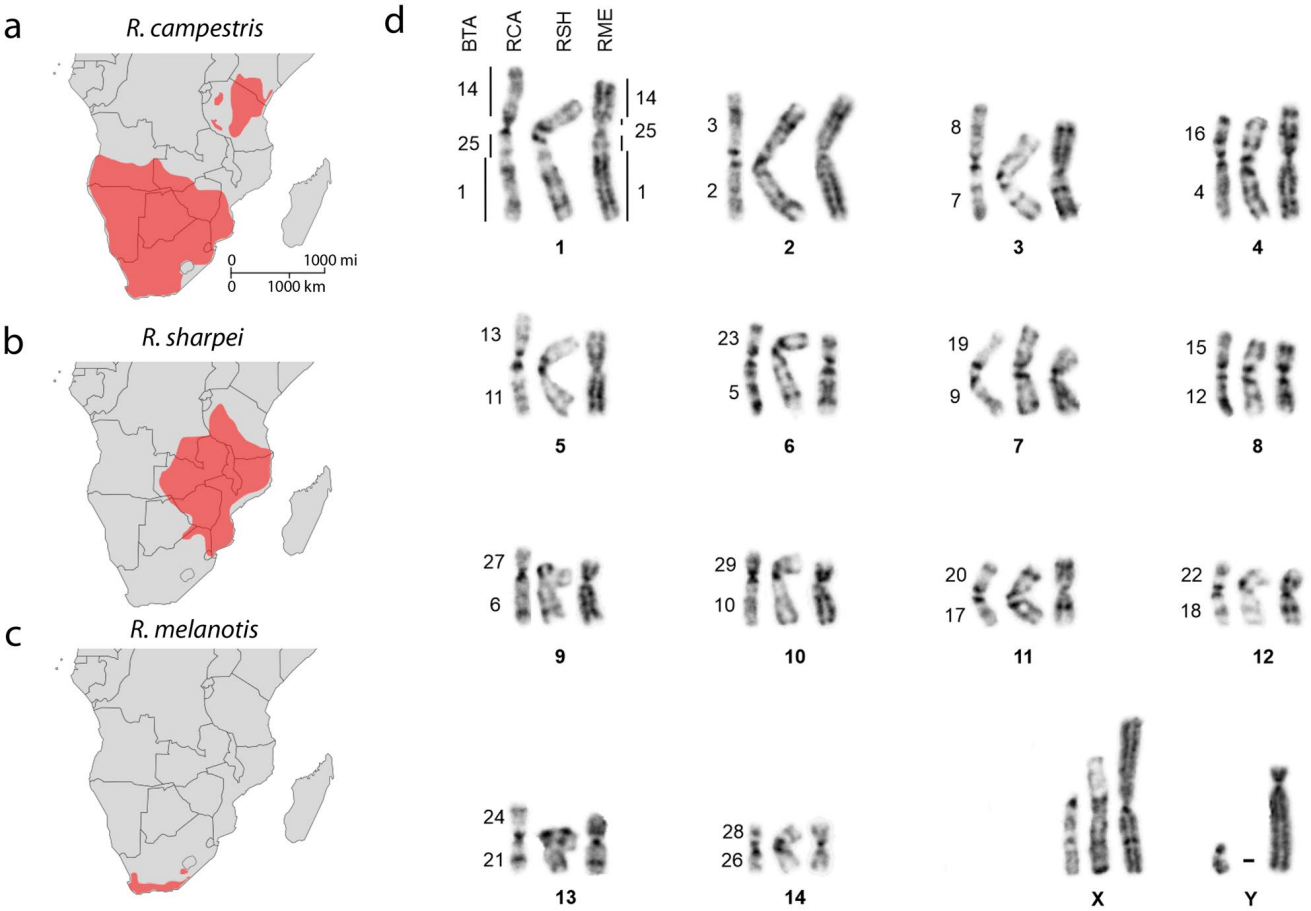
Range distribution and karyotype conservation in *Raphicerus*. (**a**) *R. campestris* (RCA) occurs in two discontinuous areas, one in East Africa and a second larger, southern African population. The two populations are separated by *Brachystegia* woodland that largely defines the distributional limits of (**b**) *R. sharpei* (RSH) which extends from Tanzania to northeast South Africa and eastern Swaziland. (**c**) *R. melanotis* (RME) is almost exclusively a Western Cape endemic largely confined to the Cape Floristic Region. Distribution maps are redrawn from^67–69^. (**d**) Comparison of the G-banded chromosomes of RCA, RSH and RME with the corresponding *Bos taurus* (BTA) syntenic relationships shown to the left in each instance.

Three species of *Raphicerus* are conventionally recognized in Africa^30^. The relatively extensive East and Southern African distributions of *R. campestris* (RCA) and *R. sharpei* (RSH) contrast sharply with *R. melanotis* (RME) (Fig. 1a–c). Morphology provides support for a closer association between RME and RSH—these include commonalities in lip, mouth and limb structure^30^ that led at times to the two “grysboks” (RSH and RME) being considered conspecific^31,32^. However, their phylogeny is conflicted in the molecular studies published to date. Initially, a sister relationship was retrieved between RCA and RSH with RME basal^33,34^ but more recently a basal RCA, with RSH and RME as sister taxa has been reported^35^. In the sections below, we examine the outcomes of our molecular cytogenetic investigation through the lens of these contrasting topologies.

### Cytogenomics of *Raphicerus* species

*Raphicerus* chromosomal relationships were analyzed in a detailed comparative study that included differential banding, comparative FISH by chromosome paints, and BAC probes. Region-specific and heterochromatic painting probes were used to analyse the X chromosome structure.

#### Autosomal syntenic regions, chromosome number and NOR location

Chromosomal syntenies between RSH, RCA, RME and *Bos taurus* (cattle, BTA) were identified by G-banding (Fig. 1d) and subsequently verified using painting probes derived from cattle. With the exceptions detailed under their molecular analysis below, the G-banded autosomes share identical banding patterns.

The diploid chromosome number of the *Raphicerus* species was 2n = 30 and comprised 14 pairs of metacentric and submetacentric autosomes that share identical Robertsonian (Rb) fusions. NORs were located on BTA orthologues 2, 3, 4, 5, 8, 16, 18 in RME, 2, 3, 4, 5, 16 in RSH and 2, 3, 4, 5 in RCA (Supplementary Fig. S1). A single derived location (BTA16) unites RME and RSH to the exclusion of RCA (BTA2, 3, 4, 5) favouring the topology retrieved by Bärmann and co-workers^35^ mentioned above. This finding underscores earlier reports of the potential usefulness of NORs as phylogenetic markers in bovids^29,36^.

**Figure 2:**
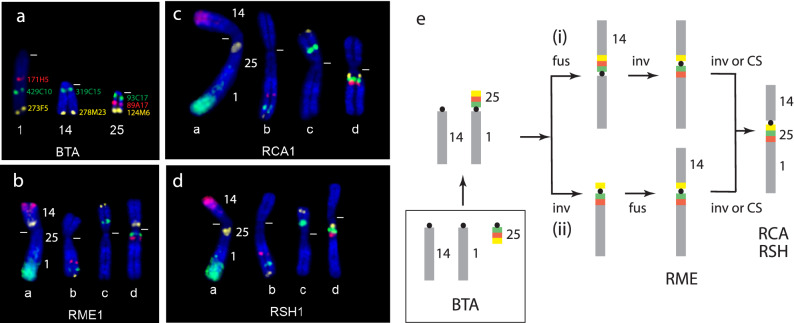
FISH analysis of *Raphicerus* chromosome 1. (**a**–**d**) Images showing the relative positions of eight BACs on BTA1, BTA14 and BTA25 that were used to orientate the corresponding orthologous segments in the *Raphicerus* species. The first chromosome in in panels **b**-**d** shows hybridization to the region-specific painting probes BTA1qd, BTA14qd and BTA25qd; grey bar indicates centromere position. (**e**) Schematic reconstruction from these data showing two possible derivations (i and ii) of this autosome (see text for details). Key: *inv* inversion, *fus* fusions, *CS* centromeric shift.

The biarmed autosomal composition of the karyotypes reflect the effects of serial Rb fusions. The only exception to this involves the largest autosomal pair (chromosome 1) present in all three *Raphicerus* species. We reconstructed the arrangement of the BTA syntenic blocks comprising this chromosome using region-specific painting probes and BACs derived from the distal ends of the corresponding BTA chromosomes (see “Material and methods”). Our region-specific painting probes showed that the q-arm is the product of a fusion between the cattle orthologs BTA25 and BTA1; the p-arm reflects a fusion between the q arm described above and the BTA14 ortholog (Fig. 2). A detailed, fine-scale analysis of this compound chromosome using BACs that mapped to the proximal (93C17), middle (89A17) and distal (124M6) portions of BTA25 (Supplementary Table S1) revealed a shared derived orientation (124M6, 93C17, 89A17) compared to cattle.

Two equally parsimonious solutions (Fig. 2e) can be hypothesized for the disrupted BTA25 synteny as evidenced by the ordering of the BACs in RME compared to its congeners, RSH and RCA. The initial amalgamation of BTA1 and BTA25 at the base of *Raphicerus* was followed by (i) its fusion with BTA14; thereafter, a pericentric inversion disrupted the BTA25 synteny as evident in RME: 124M6(Yellow)::**centromere**::93C17 (Green)::89A17(Red). The alternative explanation (ii) holds that the initial Rb1;25 fusion underwent a pericentric inversion: 124M6(Yellow)::**centromere**::93C17(Green)::89A17(Red), followed by its fusion with the BTA14 ortholog. The shared derived configuration in RCA and RSH: **centromere**::124M6(Yellow):: 93C17(Green)::89A17(Red) is attributable to either a subsequent pericentric inversion or centromeric shift in their common ancestor. This conflicts with the RME + RSH sister species association suggested by NOR placement but is consistent with the outcomes of earlier mitochondrial and nuclear DNA analyses^33,34^.

#### Characterization of centromeric heterochromatic regions

**Figure 3:**
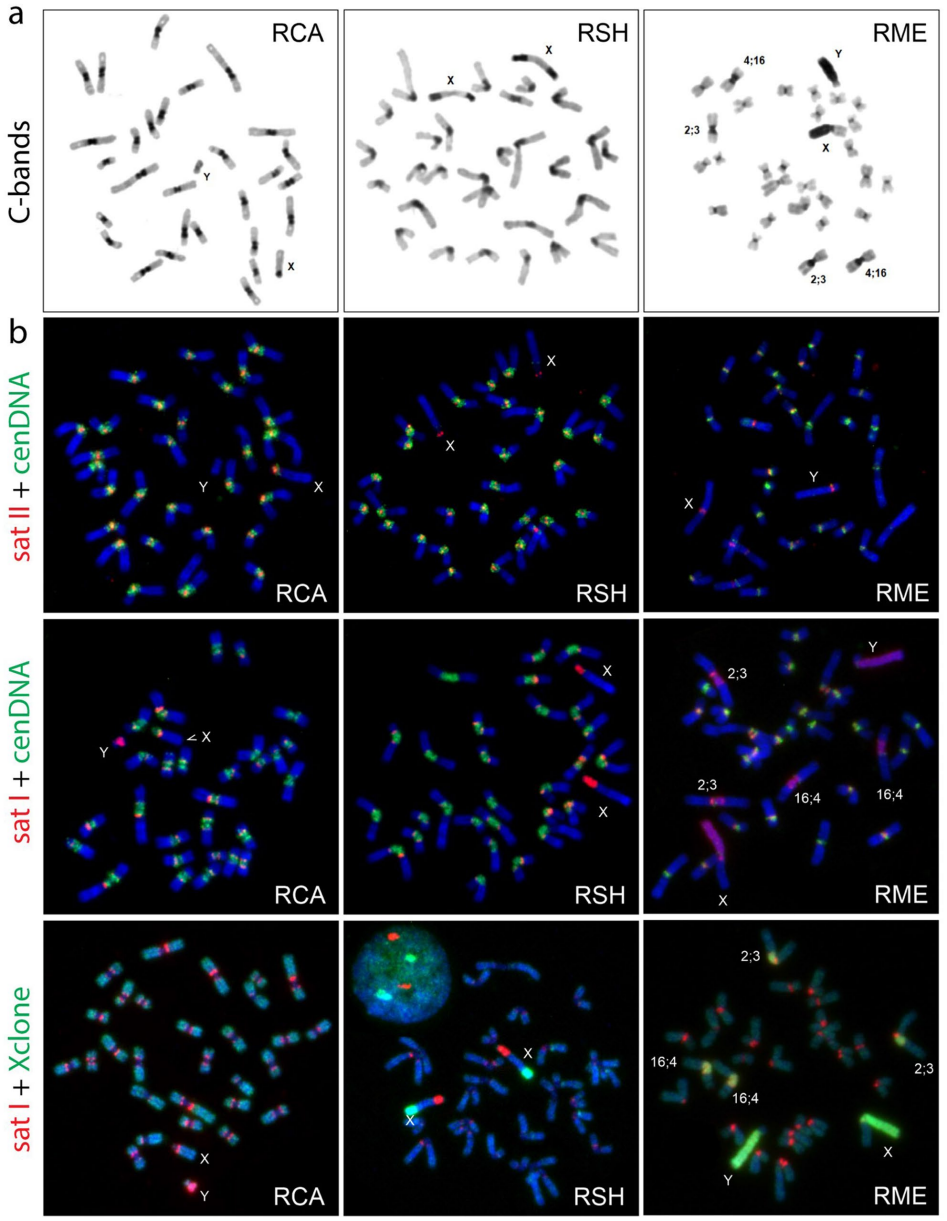
Molecular characterization of repeat sequences. (**a**) C-banding. Note the different locations of heterochromatic blocks on the X chromosomes and the relatively large amounts of pericentromeric heterochromatin in two fusion chromosomes Rb2;3 and Rb4;16 in RME. (**b**) FISH signals detected using satI, satII repeat probes and the microdissected cenDNA and Xclone probes in the three *Raphicerus* species.

C-banding showed similar amounts of autosomal centromeric heterochromatin in RCA and RSH while RME, with two exceptions discussed below (pairs 2 and 4), possesses comparatively small C-band regions (Fig. 3a). However, mere variation in pericentromeric quantity without a quantifiable measure of its abundance and a thorough understanding of the nature of the composition of the repeat sequences is problematic, posing questions of homology that confound cladistic interpretation^37,38^ and we consequently omit it as a cytogenetic character in our analyses.

#### Mapping of repetitive satellite DNA

We next explored the organization of repeats in these regions in some detail (Fig. 3b) using satI, satII and two microdissected probes, one from the centromeric region of RCA (cenDNA), the other from the RME Xp (Xclone, see “Material and methods”). The satI and satII probes targeted discrete chromosomal domains—satI sequences localized to the outer boundary of the centromeric region (pericentromeric region) and satII to the centromeric region in all species. The cenDNA probe, on the other hand, hybridized to the region between satI and satII domains. The distribution of satellite DNA into separate domains has been previously described among others, in sheep, Kirk's dik-dik and dama gazelle^39–41^ and more broadly within the Bovinae^26^.

Although the organization of the satellite fractions in the RME autosomes followed those described above there were exceptions to this pattern. These entailed the prominent C-positive pericentromeric regions of RME pairs 2 and 4 that correspond, respectively, to the cattle orthologs BTA2;3 and BTA4;16 (Fig. 1d). In these instances, entire pericentromeric regions were hybridized by satI DNA (and also the X clone prepared from the heterochromatic arm of the RMEX chromosomes). The satII probe hybridized to the centromeric region itself but cenDNA signal was not detected (Fig. 3b) by FISH suggesting its demunition and the likely expansion of satI DNA sequences in these fusions in RME.

#### Sex chromosomes

**Figure 4:**
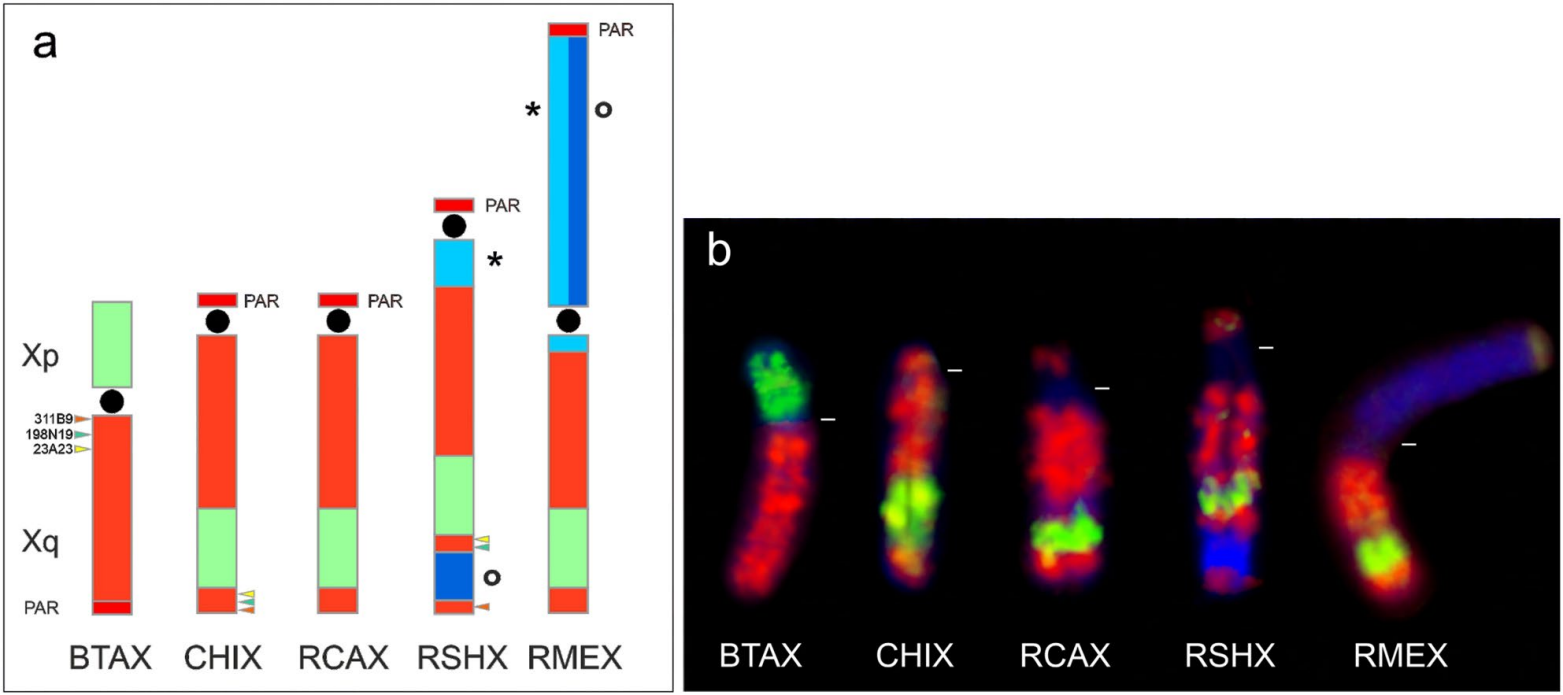
The morphology of the X chromosome differs among the *Raphicerus* species. All three show the presence of the so-called “acrocentric caprine X” (also referred to as the Suni type) with sequences orthologous to BTA Xp transposed approximately two thirds down the length of the euchromatic X chromosome^43^. (**a**) Schematic showing the relative positions of the BTA Xp (green) and BTA Xq (red) sequences and (**b**) FISH results using microdissected painting probes to this region. The distal heterochromatic block in RSH Xq was located between BACs 198N19 and 311B9. BAC 23A23 was proximal to 198N19. Key: * = satI DNA; O = SINE/LINE DNA repeats; */O = RME Xp arm comprises both satI and SINE/LINE; PAR = pseudoautosomal regions.

The *Raphicerus* X chromosomes are of the “caprine” type, the most commonly encountered X chromosome morphology within Antilopinae and considered likely to reflect the ancestral state of this chromosome for bovids in general^28,29,42–45^. G-banding (Fig. 1d), C-banding (Fig. 3a) and fine-scale FISH analyses with region-specific paints^41^ and PAR probes 302C6 and 326C13 (Supplementary material, Table S1), show that the RCAX is similar to that of the goat (CHI) included as an outgroup species in our study. It has the same acrocentric morphology, with the pseudoautosomal region located distally at the tip of the short p-arm (Fig. 4). In contrast, the morphology of the RSH and RME X chromosomes are derived compared to CHI. RSH has an acrocentric X with the PAR located at the distal end of the short arm (as with CHI); C-banding revealed two large blocks of heterochromatin located in the q arm, one proximal to the centromere and the other towards the distal end of the chromosome (Fig. 3a). The proximal block comprises satI DNA, while the distal block of heterochromatin has accumulated SINE/LINE repeats (confirmed by BLASTN searches), as shown by its strong hybridization with the X clone prepared from *R. melanotis* (Fig. 3b, Fig. 4b). Hybridization with satII gave fluorescence to the centromeric region of the chromosome. The boundary between the centromeric block and the euchromatin of the X in this species was painted by the cenDNA probe. Mapping of the RSHXq region places the distal heterochromatic block between BACs 198N19 and 311B9 (Fig. 4a).

The RME X, on the other hand, is metacentric. The p arm is entirely heterochromatic (Fig. 3a) and, as with the other species, the PAR is located at the telomeric end of the p-arm (Fig. 4). SatII DNA localized to the centromeric region of X and satI to the pericentromeric region of the q arm and the entire heterochromatic p arm. Not surprisingly, there was intense hybridization of Xp by the X clone painting probe reflecting the high number SINE/LINE repeats in this region. The cenDNA probe fluoresced weakly at the centromeric region of the X (Fig. 3b). From these data, and in spite of the derived nature of both the RSH and RME X chromosomes (with respect to both RCA and the outgroup species), there is no evidence of synapomorphic similarity to the structural changes.

We detected a clear Y chromosome dimorphism in the *Raphicerus* species for which material was available (RME and RCA). The RCA Y chromosome is a small acrocentric, with the p-arm occupied by a PAR; the entire chromosome was painted by the MKI Y probe. The satI probe hybridized strongly to the q-arm of this chromosome (Fig. 3b) whereas no signal was detected with satII or cenDNA probes. The RME Y, on the other hand, is a large acrocentric (small p-arm) with a strongly heterochromatic q-arm comprising satI DNA and SINE/LINE repeats (X clone sequences). Its centromere is occupied by satII DNA and small amounts of cenDNA (Fig. 3b). The MKI Y probe hybridized to the small p-arm and the telomeric part of Yq occupied by the PAR. The pronounced differences in the size of their Y chromosomes is also noteworthy. The increase in the size of the RME is consistent with other studies that have noted the process of accumulation of additional material in one of the sex chromosomes is invariably paralleled by the addition of material onto the other^46^.

### Appraisal of autosomal chromosomal evolutionary relationships

Comparative and molecular cytogenetic analyses highlight the conserved nature of autosomes among *Raphicerus* species. Of the various chromosomal and sub-chromosomal parameters examined, only the shared derived presence of an NOR site favours RCA (RME + RSH), with BAC mapping of the BTA25 ortholog (one of the three BTA syntenies comprising the largest *Raphicerus* autosome in each taxon) suggesting rather that RCA and RSH share a closer affinity to the exclusion of RME (due to an inversion or centromere transposition)—a single genetic character in each instance.

However, statistical modelling based on a rooted tree with three taxa^47^ shows clearly that no single genomic character can be considered definitive in providing support for a particular clade. The model predicts that, conditional on certain assumptions being met, three non-contradictory SINES (or chromosomal characters, see^48^) are required to reject an alternative phylogenetic hypothesis at the 95% confidence level. This clearly emphasizes the lack of robust support for either phylogenetic grouping in our investigation. The most probable explanation for this is the species’ rapid radiation—a view supported by transversion-based cytochrome b molecular clock calibrations^33^ that show the three lineages radiated in a narrow window ~ 2.6–1.4 mya. Divergences from a common ancestor that are close in evolutionary time would be anticipated to provide limited opportunity for the appearance of shared derived chromosomal rearrangements.

### Chromosomal speciation by X-chromosome differentiation: an hypothesis

We postulate that lineage divergence in *Raphicerus* was most probably underpinned by the fragmentation of an ancestral population with sufficient adaptability to establish subpopulations in diverse African habitats. As has been suggested^49^, the logical culmination of this is the formation of distinct allopatric species-pairs. More specifically, and as with *Raphicerus*, the distribution patterns are often along a South-East axis reflecting sequestration in refugia and subsequent expansion due to climatic oscillation in the African Pliocene/Pleistocene^49,50^. When the species extended their ranges and coalesced in areas of sympatry (i.e., RME is broadly sympatric with RCA, RCA is marginally sympatric with RSH in the northern and southern parts of the RSH range, Fig. 1a–c), genetic incompatibilities that arose during geographic isolation would have the potential to further facilitate lineage divergence.

**Figure 5:**
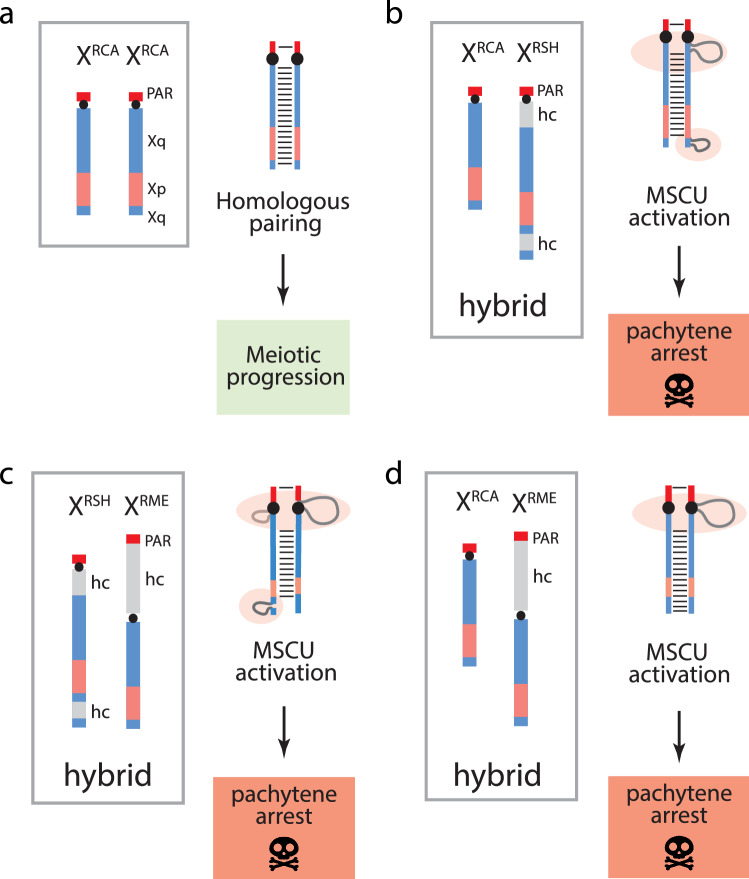
Effects of asymmetric pairing of heterochromatic (hc) blocks on meiotic progression in the homogametic sex. (**a**) Fully developed synaptonemal complex characterizing meiosis in RCA females (which possess no intercalary heterochromatin); (**b**–**d**) synapsis of heteromorphic *Raphicerus* X chromosomes, a potential outcome following interspecies hybridization showing large unsynapsed hc loops that would elicit an MSUC response in female meiosis. Red = PARs; Pink = euchromatic sequences orthologous to BTA Xp; Blue = euchromatic sequences orthologous to BTA Xq (Supplementary Fig. S2 for details).

In the light of the data presented here, we propose that lineage specific differences in location and amount of X chromosome heterochromatin (comprising repeat sequences that evolve rapidly) underpinned the divergence and subsequent recent speciation in these species. This is predicated on the expectation that female hybrids with asymmetric heterochromatic blocks would incur a selective disadvantage due to impaired meiosis resulting in sterility or reduced gamete production (Fig. 5)—i.e., they serve as potentially important but infrequently studied contributors to postzygotic isolation.

In fact, a major mechanistic challenge in meiosis requires homologous chromosomes to pair and to establish connections between them. This has led to an intricate system of signalling mechanisms as part of a complex surveillance network, the Meiotic Checkpoint Network (MCN), that is necessary to ensure the faithful progression of meiosis^51^. One checkpoint in the surveillance network detects the presence of partial, or completely unsynapsed regions during prophase I which, if triggered, induces transcriptional repression known as Meiotic Silencing of Unsynapsed Chromatin (MSUC)—an epigenetic silencing programme conserved in mammals^52–55^. MSUC is characterised by an accumulation of chromatin modifications (i.e., histone H2AX phosphorylation, γH2AX) in response to asynapsed chromatin and unrepaired DSBs during prophase I^52,53^. If unsynapsed regions persist, this results in meiotic arrest and finally apoptosis, impacting individual fitness.

Following the premise of meiotic silencing (MSUC) outlined above, it is reasonable to expect that the heterochromatic regions distinguishing the RME and RSH X chromosomes (which are both substantial in size, differ in satellite composition and do not align on the respective chromosomes) would be particularly problematic for hybrid female meiosis should it occur. Meiosis in male hybrids, on the other hand, would not be affected as X–Y pairing through the PAR is not jeopardised. The asynapsed regions of the Xs would be expected to present as one large, unpaired heterochromatic loop in RCA x RME hybrids, two in RCA x RSH hybrids (one proximal and one distal) and, although currently geographically distinct, three in the case of RME x RSH hybrids (Fig. 5). In each instance, meiosis would be compromised due to the activation of the MSUC checkpoint resulting in a selective disadvantage in females due to impaired meiosis, sterility or reduced gamete production that could lead to co-evolution within populations and divergence between the *Raphicerus* lineages. A caveat to this hypothesis concerns the triggering of MSUC in instances involving heterochromatic block polymorphisms within species. Although we cannot directly address this with our data, a probable explanation may be that the MSUC response depends on the size of mismatched heterochromatic blocks. Misalignment resulting from relatively small, incremental changes to the size of repeat arrays may escape MSUC allowing for the gradual alteration in block sizes within lineages and no impairment of fertility. It is only when these are substantive enough that a meiotic checkpoint response is elicited.

Although few reports are available on the meiotic behaviour of heterochromatic regions in mammalian X chromosomes, support for our hypothesis is indirect and based on female meiosis in species that show pronounced inter- and intraspecific X chromosome heteromorphisms. One such example involves the hamster (*Mesocricetus auratus*) and the short-tailed bandicoot rat (*Nesokia indica*), rodents with large quantities of heterochromatin on their X chromosomes^56^. Asynapsis of heterochromatic regions was observed in a substantial number of oocytes in these species, whereas the X chromosomes in the mouse (*Mus dunni*) and lesser bandicoot rat (*Bandicota bengalensis*), which present little heterochromatin, were fully synapsed. These findings were subsequently extended in the field vole (*Microtus agrestis*), where pairing of its richly heterochromatic X chromosomes is ephemeral and terminates precociously in pachytene and metaphase I^46^. These data suggest that size of the heterochromatic region is important and can affect synapsis in various ways—through delayed synapsis, precocious separation, or by asynapsis^56,57^.

## Conclusions

There is now a large, contemporary body of evidence to suggest that genetic conflict, drive (various manifestations) and differences in the sequence, amount and proteins involved in the epigenetic modification of heterochromatin, are potential sources of chromosomal novelty that may result in post-zygotic isolation and incipient species divergence^7,20,21,53,58,59^. To this we add the activation of the meiotic checkpoint signalling network due to the compromised pairing of asymetric heterochromatic blocks in the homogametic sex. By acting as an additional evolutionary constraint on reproductive compatibility, it serves chromosomal speciation. Asynapsed chromosomal segments (euchromatic as well as heterochromatic) elicit its activation, the transcriptional silencing of unsynapsed chromatin and, through meiotic arrest and the checkpoint-dependent induction of apoptotic cell death^51^, post-zygotic isolation. Building support for a direct, mechanistic link between the asynapsis of large heterochromatic blocks and female hybrid sterility (the converse of the Haldane effect) will depend on a deeper understanding of the connection between them, as well as the processes that have shaped genomic and sex chromosome evolutionary patterns within a more diversified taxonomic base.

## Materials and methods

### Source of material, cell culture and banding techniques

Metaphase chromosomes of *Raphicerus sharpei* (female; RSH – commonly known as Sharpe’s Grysbok), *R. campestris* (male; RCA—Steenbok) and *R*. *melanotis* (male; RME—Cape Grysbok), the three recognized species of the genus, were derived from cryopreserved fibroblast cell lines cultured in Dulbecco’s Modified Eagle Medium (DMEM, Gibco) under standard conditions. In the case of *Bos taurus* (BTA, female) and *Capra hircus* (CHI, female), used as outgroups for the cytogenetic study, chromosome preparations were made from phytohemagglutinin-stimulated lymphocyte cultures of whole blood grown in RPMI 1640 medium (Sigma-Aldrich). Culture protocols and the differential staining of chromosomes (GTG- and C-banding) followed conventional techniques^60–62^. The G-banded chromosomes of RCA, RSH and RME were numbered in accordance with the BTA standard^63^. All experimental protocols, sample collection and the processing of material was approved by the Stellenbosch University’s Research Ethics Committee (ethics no. SU-ACUD15-00103) and performed in accordance with relevant guidelines and regulations.

### DNA probes and fluorescence in situ hybridization (FISH)

#### Whole and subchromosomal painting probes

Whole chromosome painting probes from cattle (BTA1-29) were used for cross-species hybridization to RSH, RCA and RME. The orientation of the syntenic blocks comprising RSH1, RCA1 and RME1 (the largest fusion chromosome in *Raphicerus*) was by region-specific paints BTA25qd, BTA14qd and BTA1qd. Analysis of the X chromosomes relied on arm- and region-specific painting probes from cattle and goat^41^ that localized to BTAXp, BTAXq, BTA Xq 3.6-qter and CHI Xq 4.1-qter respectively. Y chromosomes were examined using a painting probe originally prepared from Kirk´s dik-dik (*Madoqua kirkii*, MKI^40^). Detection of Nucleolar Organizer Regions (NORs) was by FISH probes prepared from the antelope, *Nanger dama*^41^.

In all instances painting probes were prepared by laser microdissection (PALM Microlaser system, Carl Zeiss MicroImaging GmbH, Munich, Germany) and DNA amplified by degenerate oligonucleotide primed polymerase chain reaction (DOP-PCR: primer sequence CCGACTCGAGNNNNNNATGTGG)^64^. Labelling during the secondary PCR^64^ was with Orange-d UTP or Green-dUTP (Abbott, IL, USA).

#### BAC clones

A panel of 13 BACs were chosen from the CHORI-240 cattle library on the basis of the NCBI Primary Assembly ARS-UCD1.2 and obtained from the BACPAC Resource Center, Children’s Hospital Oakland Research Institute (Supplementary material, Table S1). Of these, eight provided resolution on the orientation of BTA syntenies (BTA1, BTA14, BTA25) in the largest autosomal chromosome in the *Raphicerus* karyotypes. Two (302C6, 326C13), from the pseudoautosomal region (PAR) of BTAX, detect the corresponding regions in *Raphicerus* and three (311B9, 198N19, 23A23), also from BTAX, were selected to localize the heterochromatic blocks in the distal part of the *R. sharpei* X chromosome (see Supplementary Table S1 for details). BAC clones were labelled with biotin-16-dUTP or digoxigenin-11-dUTP as specified in the BioPrime Array CGH Genomic Labelling Module (Invitrogen, Carlsbad, CA, USA) and detected by Avidin-CY3 (Amersham Pharmacia Biotech, NJ, USA) and antidigoxigenin-fluorescein (Roche Mannheim, Germany).

#### X clone prepared from R. melanotis

Microdissection was used for the isolation of the Xp arm of this species which, on C-banding (see Results and discussion), is entirely heterochromatic. DNA amplification of the microdissected chromosome was performed using the manufacturer’s instructions for the GenomePlex Single Cell Whole Genome Amplification Kit (Sigma-Aldrich, Taufkirchen, GE) and checked by FISH. Amplicons were ligated into a pDrive vector (Qiagen, Hilden, Germany). Sixty clones were screened by DOT-BLOT hybridization^65^; eight were initially chosen on intensity and subsequently fluorescently labelled by Green-dUTPs and hybridized back to RME chromosomes. Three FISH-positive clones were sequenced (Sanger sequencing) and used for cross-species hybridization to RSH and RCA. Sequences were deposited in GenBank under accession numbers MW133064, MW133065, MW133066 and compared to those in the GenBank database using BLASTN searches.

#### Centromeric probes

The analysis of the centromeric DNA composition and its organization within *Raphicerus* species was done using satI, satII and cenDNA probes. The *Eudorcas thomsoni* satellite I clone^65^ was used for detection of satI sequences (NCBI accession number KF787949; 784 bp in length). These sequences are related to the well-documented 1.714 satI DNA family. The satII probe (accession number KM111601, 563 bp in length) was originally prepared from *E. thomsoni*^66^ and is related to 1.723 satII DNA family. Sat DNA sequences were labelled by Orange-d UTP or Green-dUTP (Abbott, IL, USA).

For generation of the cenDNA probe, DNA templates were taken from centromeric regions of *R. campestris* by laser microdissection, amplified using the GenomePlex WGA Kit and cloned. Positive clones were selected by DOT-BLOT hybridization and checked by FISH to *R. campestris* chromosomes. A single 241 bp clone, comprised almost exclusively of the trinucleotide repeat AAG (99%), was chosen on FISH intensity. Its sequences were compared to those in GenBank database using BLASTN searches, deposited in GenBank under accession number MW133067 and subsequently hybridized to *R. melanotis* and *R. sharpei* to analyse their FISH patterns.

FISH protocols for satellite detection, chromosome painting and the use of BAC probes followed^41,66^. Hybridization signals were examined using Zeiss Axio imager.Z2 fluorescence microscope with appropriate fluorescent filters; image capture was by a CoolCube CCD camera (MetaSystems, Altlussheim, Germany) and image analysis by ISIS (MetaSystems).

### Ethics declaration

Sample collection and the processing of material was authorized under Stellenbosch University’s ethics no. SU-ACUD15-00103.

## Supplementary Information

Supplementary Information

## Data Availability

Sequences were deposited in GenBank under the following accession numbers: X clones—MW133064 (clone 1), MW133065 (clone 2), MW133066 (clone 3); cenDNA clone—MW133067. Datasets supporting this article have been uploaded as part of the supplementary material.
