# Supplementary material for: Chromosomal evolution in Raphicerus antelope suggests divergent X chromosomes may drive speciation through females, rather than males, contrary to Haldane's rule
Source: Sci Rep. 2021 Feb 4;11:3152. doi: 10.1038/s41598-021-82859-0 (PMC7862234; doi:10.1038/s41598-021-82859-0)

Chromosomal evolution in *Raphicerus* antelope suggests divergent X chromosomes may drive speciation through females, rather than males, contrary to Haldane's rule

Terence J Robinson[1*], Halina Cernohorska[2], Svatava Kubickova[2], Miluse Vozdova[2], Petra Musilova[2], Aurora Ruiz-Herrera[3,4]

[1]Department of Botany and Zoology, Stellenbosch University, South Africa (*Address for correspondence; email tjr@sun.ac.za)
[2]Veterinary Research Institute, Hudcova 70, Brno, Czech Republic
[3]Departament de Biologia Cel·lular, Fisiologia i Immunologia, Universitat Autònoma de Barcelona (UAB), Cerdanyola del Vallès, 08193, Spain
[4]Genome Integrity and Instability Group, Institut de Biotecnologia i Biomedicina (IBB), Universitat Autònoma de Barcelona (UAB), Cerdanyola del Vallès, 08193, Spain

Supplementary Table S1: A panel of 13 BACs were used for the orientation of the BTA syntenies comprising the largest *Raphicerus* autosome, the detection of the PARs and the localization of the heterochromatic blocks in the distal part of the *R. sharpei* X chromosome. Location of BACs taken from the cattle reference genome: *Bos taurus* (assembly ARS-UCD1.2)

Chromosome location	BAC FISH probe	Location (Mb)
BTA1	171H5	58.53-58.71
BTA1	429C10	106.13-106.33
BTA1	273F5	152.62-152.81
BTA14	319C15	0.88-1.07
BTA14	278M23	81.18-81.38
BTA25	93C17	3.87-4.10
BTA25	89A17	21. 27-21.51
BTA25	124M6	37.23-37.44
BTAX PAR	302C6	138.68-138.85
BTAX PAR	326C13	137.12-137.32
BTAX	311B9	45.22-45.42
BTAX	198N19	50.79-50.96
BTAX	23A23	57.92-58.08

Supplementary Figure S1: The detection of Nucleolar Organizer Regions (NORs) in the *Raphicerus* spp.   These were located on BTA orthologues 2, 3, 4, 5, 8, 16, 18 in RME, 2, 3, 4, 5, 16 in RSH and 2, 3, 4, 5 in RCA. A single derived location (BTA16) unites RME and RSH to the exclusion of RCA (BTA2, 3, 4, 5).

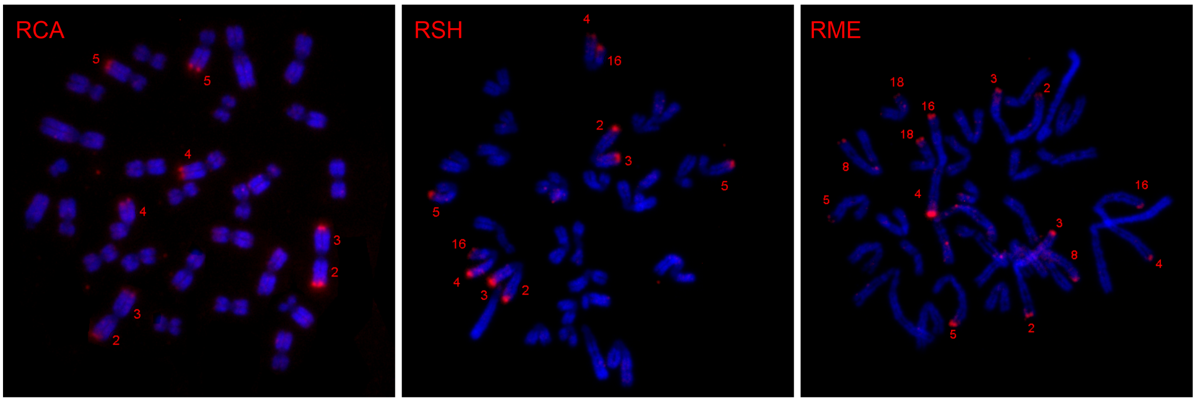

Supplement: Supplementary file 1 — Supplementary Information [file 41598_2021_82859_MOESM1_ESM.pdf]
